# Calculations for deep inelastic scattering using fast interpolation grid techniques at NNLO in QCD and the extraction of $\alpha _{\mathrm {s}}$ from HERA data

**DOI:** 10.1140/epjc/s10052-019-7351-x

**Published:** 2019-10-14

**Authors:** D. Britzger, J. Currie, A. Gehrmann-De Ridder, T. Gehrmann, E. W. N. Glover, C. Gwenlan, A. Huss, T. Morgan, J. Niehues, J. Pires, K. Rabbertz, M. R. Sutton

**Affiliations:** 1 Max-Planck-Institut für Physik, Föhringer Ring 6, 80805 Munich, Germany; 2 Institute for Particle Physics Phenomenology, Durham University, Durham, DH1 3LE UK; 3 Institute for Theoretical Physics, ETH, Wolfgang-Pauli-Strasse 27, 8093 Zurich, Switzerland; 4 Physik-Institut, Universität Zürich, Winterthurerstrasse 190, 8057 Zurich, Switzerland; 5 Department of Physics, The University of Oxford, Oxford, OX1 3PU UK; 6 Theoretical Physics Department, CERN, 1211 Geneva 23, Switzerland; 7 CFTP, Instituto Superior Técnico, Universidade de Lisboa, 1049-001 Lisbon, Portugal; 8 LIP, Avenida Professor Gama Pinto 2, 1649-003 Lisbon, Portugal; 9 Institut für Experimentelle Teilchenphysik (ETP), Karlsruhe Institute of Technology (KIT), Wolgang-Gaede-Str. 1, 76131 Karlsruhe, Germany; 10 Department of Physics and Astronomy, The University of Sussex, Brighton, BN1 9RH UK

## Abstract

The extension of interpolation-grid frameworks for perturbative QCD calculations at next-to-next-to-leading order (NNLO) is presented for deep inelastic scattering (DIS) processes. A fast and flexible evaluation of higher-order predictions for any a posteriori choice of parton distribution functions (PDFs) or value of the strong coupling constant is essential in iterative fitting procedures to extract PDFs and Standard Model parameters as well as for a detailed study of the scale dependence. The APPLfast project, described here, provides a generic interface between the parton-level Monte Carlo program NNLOjet and both the APPLgrid and fastNLO libraries for the production of interpolation grids at NNLO accuracy. Details of the interface for DIS processes are presented together with the required interpolation grids at NNLO, which are made available. They cover numerous inclusive jet measurements by the H1 and ZEUS experiments at HERA. An extraction of the strong coupling constant is performed as an application of the use of such grids and a best-fit value of $\alpha _{\mathrm {s}} (M_{{\mathrm {Z}}}) = 0.1170\,(15)_\text {exp}\,(25)_\text {th}$ is obtained using the HERA inclusive jet cross section data.

## Introduction

Modern calculations of higher-order corrections in perturbative QCD for predictions of cross sections from collider experiments are computationally very demanding. In particular, complicated measurement functions and fiducial phase-space definitions associated with differential cross sections prevent an analytic integration over the final-state kinematics, thus calling for numerical approaches. Next-to-next-to-leading order computations for differential cross-section predictions, for example, often require $\mathcal {O}(10^5)$ CPU hours due to the complicated singularity structure of the real-emission amplitudes and the delicate numerical cancellations they entail. Further challenges arise from the requirement of high precision for important benchmark processes. Common examples are jet production cross sections in both electron–proton collisions or ${\mathrm {p}} {\mathrm {p}}$ collisions, the Drell–Yan production of ${\mathrm {Z}}$ and ${\mathrm {W}}$ bosons, and gauge-boson production in association with jets.

The NNLOjet program [1] is a recent and continuously developing framework for the calculation of fully differential cross sections for collider experiments. It includes a large number of processes calculated at NNLO in perturbative QCD, implemented in a unified and holistic manner.

For a detailed study of NNLO predictions and the estimation of theoretical uncertainties, these calculations must be repeated with different input conditions. This includes, for example, using different values for the strong coupling $\alpha _{\mathrm {s}} (M_{{\mathrm {Z}}})$, different parametrisations for the PDFs, or different choices for the factorisation or renormalisation scales. Computationally even more demanding are fits for the determination of the strong coupling constant and the parton densities in the proton.

In such fits, comparisons must be performed between the data and the NNLO predictions for the many thousands of points that are drawn from the multidimensional parameter space used in the minimisation. As such, it is computationally prohibitive to run the full calculation at NNLO for each required input condition encountered in such a fit. Applications of this nature therefore critically require an efficient approach to perform the convolution of the partonic hard scattering with PDFs, change the value of the strong coupling constant, and vary the scales.

The technique of using a grid to store the perturbative coefficients stripped of the parton luminosity and factors of the strong coupling constant $\alpha _{\mathrm {s}}$, during the full Monte Carlo integration allows the convolution with arbitrary PDFs to be performed later with essentially no additional computational cost. Variation of $\alpha _{\mathrm {s}} (M_{{\mathrm {Z}}})$, and the renormalisation and factorisation scales is also possible. The grid technique, used in Ref. [2], is implemented independently in the APPLgrid [3, 4] and fastNLO [5, 6] packages. The technique works by using interpolation functions to distribute each single weight from the *x* and $\mu ^2$ phase space of the integration, over a number of discrete *a priori* determined nodes in that phase space along with the relevant interpolating function coefficients. Subsequently summing over those discrete nodes will therefore reproduce the original value for the weight, or any product of the weight with some function of the phase space parameters for that specific phase space point. One dimension in the grid is required for each parameter upon which the subsequently varied parameters will depend. For instance, for DIS processes, a dimension for *x* and $\mu ^2$ will be required. For ${\mathrm {p}} {\mathrm {p}}$ collisions, a third dimension must be added to account for the momentum fraction $x_2$ of the second proton.

This paper describes developments in the APPLfast project which provides a common interface for the APPLgrid and fastNLO grid libraries to link to the NNLOjet program for the calculation of the perturbative coefficients. The generation and application of interpolation grids for DIS jet production at NNLO [7, 8] is discussed. Grids are made publicly available on the ploughshare website [9]. A subset of these grids have previously been employed for a determination of the strong coupling constant, $\alpha _{\mathrm {s}} (M_{{\mathrm {Z}}})$ [10]. Here, additional details of the grid methodology for DIS are discussed, together with the NNLO extraction of $\alpha _{\mathrm {s}} (M_{{\mathrm {Z}}})$ using data on inclusive jet production from both H1 and ZEUS.

## DIS at NNLO and the NNLOjet framework

Jet production in the neutral-current DIS process proceeds through the scattering of a parton from the proton with a virtual photon or Z boson that mediates the interaction. The cross section for this process is given by the convolution of the parton distribution function with the partonic hard-scattering cross section

1 $$\begin{aligned} \sigma&= \int \mathrm {d}x \; f_a(x,\mu _{\mathrm{F}}) \; \mathrm {d}\hat{\sigma }_a(x,\mu _{\mathrm{R}},\mu _{\mathrm{F}}), \end{aligned}$$

which includes an implicit summation over the index *a* which denotes the incoming parton flavour. In perturbative QCD, the hard-scattering cross section can be expanded in the coupling constant

$$\begin{aligned} \mathrm {d}\hat{\sigma }_a(x,\mu _{\mathrm{R}},\mu _{\mathrm{F}})&= \sum _{p} \left( \frac{\alpha _{\mathrm {s}} (\mu _{\mathrm{R}})}{2\pi }\right) ^{k+p} \mathrm {d}\hat{\sigma }^{(p)}_a(x,\mu _{\mathrm{R}},\mu _{\mathrm{F}}) \, , \end{aligned}$$

where *k* corresponds to the power in $\alpha _{\mathrm {s}}$ at leading order (LO). Jet cross section measurements in DIS commonly employ a reconstruction in the Breit frame of reference, in which the proton and the gauge boson of virtuality $Q^{2}$ collide head-on. This is further assumed in the remainder of this work. As a consequence, jet production proceeds through the basic scattering processes $\gamma ^*g\rightarrow q\bar{q}$ and $\gamma ^*q\rightarrow qg$, thus requiring at least two partons in the final state. This choice not only gives a direct sensitivity to $\alpha _{\mathrm {s}}$ ($k=1$) but also a rare handle on the gluon density already at LO.

Calculations at higher orders in perturbation theory comprise distinct parton-level ingredients that may involve additional loop integrations and real emission. For jet production in DIS at NNLO ($p=2$), three types of contributions enter the calculation: The double-real (RR) contribution comprising tree-level amplitudes with two additional partons in the final state [11–13], the real–virtual (RV) contribution that requires one-loop amplitudes with one additional emission [14–17], and the double-virtual (VV) contribution involving two-loop amplitudes [18–20]. Each of these ingredients are separately infrared divergent and only finite after taking their sum, as dictated by the Kinoshita–Lee–Nauenberg theorem. The different manifestations of the singularities among the three contributions, related to the distinct parton multiplicities, makes the cancellation of infrared singularities a highly non-trivial task. Fully differential predictions in particular, require a procedure to re-distribute and cancel the singularities while retaining the information on the final-state kinematics. The antenna subtraction formalism [21–23] accomplishes this by introducing local counter terms with the aim to render each contribution manifestly finite and thus amenable to numerical Monte Carlo integration methods. The partonic hard-scattering cross section can be schematically written as

2 $$\begin{aligned} \int \mathrm {d}\hat{\sigma }^{(2)}_a&= \int _{\varPhi ^{(n+2)}} \Bigl ( \mathrm {d}\hat{\sigma }^{RR}_a - \mathrm {d}\hat{\sigma }^{S}_a \Bigr ) +\int _{\varPhi ^{(n+1)}} \Bigl ( \mathrm {d}\hat{\sigma }^{RV}_a - \mathrm {d}\hat{\sigma }^{T}_a \Bigr ) \nonumber \\ {}&\quad +\int _{\varPhi ^{(n)}} \Bigl ( \mathrm {d}\hat{\sigma }^{VV}_a - \mathrm {d}\hat{\sigma }^{U}_a \Bigr ) \, , \end{aligned}$$

where the subtraction terms $\mathrm {d}\hat{\sigma }^{S,T,U}_a$ absorb in their definition the NNLO mass-factorisation terms from the PDFs and are explicitly given in Ref. [8]. Note that differential distributions can be accommodated in Eq. (1) via event selection cuts in the measurement functions that are implicitly contained in $\mathrm {d}\hat{\sigma }^X_a$.

The NNLOjet framework [1] provides the necessary infrastructure to perform calculations at NNLO using the antenna subtraction method following the master formula (2) and incorporates all available processes under a common code base. The parton-level Monte Carlo generator evaluates the integral for each perturbative order ($p=0,\ldots$) by summing over samples of the phase space $(x_m,\varPhi _m)_{m=1,\ldots ,M_p}$ with their associated weights $w^{(p)}_{a;m}$. The cross section in Eq. (1) can then be computed via

3 $$\begin{aligned} \sigma&\xrightarrow {\text {MC}} \sum _{p} \sum _{m=1}^{M_p} \; \left( \frac{\alpha _{\mathrm {s}} (\mu _{\mathrm{R}} {}_{;m})}{2\pi }\right) ^{k+p} \nonumber \\&\quad \times f_{a}(x_m, \mu _{\mathrm{F}} {}_{;m}) \; w^{(p)}_{a;m} \; \mathrm {d}\hat{\sigma }^{(p)}_{a;m} \, , \end{aligned}$$

using the short-hand notation

$$\begin{aligned} \mu _{X;m}&\equiv \mu _X(\varPhi _m) \quad \text {for } X=\mathrm {R},\,\mathrm {F}, \\ \mathrm {d}\hat{\sigma }^{(p)}_{a;m}&\equiv \mathrm {d}\hat{\sigma }^{(p)}_{a}(x_m,\mu _{\mathrm{R}} {}_{;m},\mu _{\mathrm{F}} {}_{;m}). \end{aligned}$$

For the interface of the NNLOjet code to the grid-filling tools described in Sect. 3, additional hook functions are provided that, e.g., allow for a full decomposition of the differential cross section $\mathrm {d}\hat{\sigma }^{(p)}_{a}$ into the coefficients of the logarithms in the renormalisation and factorisation scales:

4 $$\begin{aligned}&\mathrm {d}\hat{\sigma }^{(p)}_{a} (\mu _{\mathrm{R}} ^2,\mu _{\mathrm{F}} ^2) = \sum _{{\begin{array}{c} \alpha , \beta \\ \alpha +\beta \le p \end{array}}} \mathrm {d}\hat{\sigma }^{(p | \alpha ,\beta )}_{a} \ln ^{\alpha }\left( \frac{\mu _{\mathrm{R}} ^2}{\mu ^2}\right) \ln ^{\beta } \left( \frac{\mu _{\mathrm{F}} ^2}{\mu ^2}\right) \, , \end{aligned}$$

where $\mu$ is the reference scale of the decomposition. This ensures maximal flexibility for the interface to accommodate different prescriptions, such as the different strategies pursued by APPLgrid and fastNLO for the reconstruction of the scale dependence.

## The APPLgrid and fastNLO packages

The grid technique allows an accurate approximation of a continuous function *f*(*x*) to be obtained from the knowledge of its value at discrete nodes $a\equiv x^{[0]}< x^{[1]}< \ldots < x^{[N]}\equiv b$ that partition the interval $[x_{\mathrm{min}},x_{\mathrm{max}}]$ into *N* disjoint sub-intervals. To this end, interpolation kernels $E_i(x)$ are introduced for each node *i*, which are constructed from polynomials of degree *n* and satisfy $E_i(x^{[j]})=\delta _i^j$. The set of interpolation kernels further form a partition of unity,

5 $$\begin{aligned} 1&= \sum _{i=0}^{N} E_i(x) \quad \text {for }a \le x \le b. \end{aligned}$$

As a result, the continuous function *f*(*x*) can be approximated as

6 $$\begin{aligned} f(x)&\simeq \sum _{i=0}^{N} f^{[i]} \; E_i(x) \quad \text {with } f^{[i]} \equiv f(x^{[i]}) . \end{aligned}$$

In practice, the interpolation is often set up using equidistant nodes ($x^{[k]}=x^{[0]}+k\,\delta x$) for simplicity. This can however result into a sub-optimal placement of grid nodes resulting in a poor interpolation quality, which in turn would require an increase in the number of nodes to achieve the required target accuracy. Alternatively, the accuracy can be greatly improved by performing a variable transformation $x \longmapsto y(x)$ that increases the density of nodes in regions where *f*(*x*) varies more rapidly. In this case, nodes are chosen with respect to *y*(*x*) and the corresponding interpolation kernels are denoted by $E^y_i(x)$.

Finally, when the function *f*(*x*) appears under an integral, the integration can be approximated by a sum over the nodes *i*,

7 $$\begin{aligned}&\int _a^b \mathrm {d}x \; f(x) \; g(x) \simeq \sum _{i=0}^{N} f^{[i]} \; g_{[i]} \, , \end{aligned}$$

using the definition

8 $$\begin{aligned} g_{[i]}&\equiv \int _a^b \mathrm {d}x \; E_i(x) \; g(x) \, . \end{aligned}$$

The time-consuming computation of the integral can then be performed once and for all to produce a grid $g_{[i]}$ ($i=0,\ldots ,N$) and the integral in Eq. (7) can be approximated for different functions *f*(*x*) using the sum from the right hand side, which can be evaluated very quickly.

### Application to the DIS cross section

For DIS processes, the different parton densities $f_a(x,\mu _{\mathrm{F}})$ can be included using the grid technique. In this case, a two-dimensional grid in the two independent variables *x* and $\mu _{\mathrm{F}}$ is constructed. The respective interpolation kernels $E^y_i(x)$ and $E^\tau _j(\mu _{\mathrm{F}})$ can be chosen independently for the two variables, introducing the additional transformation in the scale variable, $\mu _{\mathrm{F}} \longmapsto \tau (\mu _{\mathrm{F}})$. Typical transformations for DIS are for instance

9 $$\begin{aligned} y(x) = \ln \frac{1}{x} + \alpha (1 - x ) \quad \text {or}\quad y(x)= \ln ^\alpha \frac{1}{x} \end{aligned}$$

for the momentum fraction, and

10 $$\begin{aligned} \tau (\mu ) = \ln \ln \frac{\mu ^2}{\varLambda ^2} \quad \text {or}\quad \tau (\mu ) = \ln \ln \frac{\mu }{\varLambda }, \end{aligned}$$

for the hard scale, where the parameter $\alpha$ can be used to increase the density of nodes at high or low values of *x* or $\mu$, and $\varLambda$ can be chosen of the order of $\varLambda _{\mathrm {QCD}}$, but need not necessarily be identical. Additional transforms are available in both APPLgrid and fastNLO.

For any value of *x* and $\mu$, both the PDFs and the running of the strong coupling can then be represented by a sum over the interpolation nodes,

11 $$\begin{aligned} \alpha _{\mathrm {s}} (\mu ) \; f_a(x,\mu ) \simeq \sum _{i,j} \alpha _{\mathrm {s}} ^{[j]} \; f^{[i,j]}_{a} \; E^y_i(x) \; E^\tau _j(\mu ), \end{aligned}$$

where $\mu _{\mathrm{R}} =\mu _{\mathrm{F}} \equiv \mu$ has been set for simplicity. The computationally expensive convolution with the PDFs from Eq. (1), which further includes an implicit phase-space dependence through the scale $\mu$, can thus be approximated by a two-fold summation,

12 $$\begin{aligned} \sigma&= \sum _{p} \int \mathrm {d}x \left( \frac{\alpha _{\mathrm {s}} (\mu )}{2\pi }\right) ^{k+p} f_a(x,\mu ) \; \mathrm {d}\hat{\sigma }^{(p)}_a(x,\mu ) \nonumber \\ {}&\simeq \sum _{p} \sum _{i,j} \biggl (\frac{\alpha _{\mathrm {s}} ^{[j]}}{2\pi }\biggr )^{k+p} f^{[i,j]}_{a} \; \hat{\sigma }^{(p)}_{a[i,j]} \, . \end{aligned}$$

Here, the grid of the hard coefficient function at the perturbative order *p* has been defined as

13 $$\begin{aligned} \hat{\sigma }^{(p)}_{a[i,j]}&= \int \mathrm {d}x \; E^y_i(x) \; E^\tau _j(\mu ) \; \mathrm {d}\hat{\sigma }^{(p)}_a(x,\mu ) \, , \end{aligned}$$

which can be readily obtained during the Monte Carlo integration as described in Eq. (3) by accumulating the weights

14 $$\begin{aligned} \hat{\sigma }^{(p)}_{a[i,j]}&\xrightarrow {\text {MC}} \sum _{m=1}^{M_p} E^y_i(x_m) \; E^\tau _j(\mu _{m}) \; w^{(p)}_{a;m} \; \mathrm {d}\hat{\sigma }^{(p)}_{a;m} \end{aligned}$$

during the computation.

### Renormalisation and factorisation scale dependence

With the hard coefficients $\hat{\sigma }^{(p)}_{a[i,j]}$ determined separately order by order in $\alpha _{\mathrm {s}}$, it is straightforward to restore the dependence on the renormalisation scale, $\mu _{\mathrm{R}}$, and factorisation scale, $\mu _{\mathrm{F}}$, using the RGE running of $\alpha _{\mathrm {s}}$ and the DGLAP evolution for the PDFs. To this end, any functional form can be chosen that depends on the scale $\mu$ that was used during the grid generation (14);

15 $$\begin{aligned} \mu _X&= \mu _X(\mu ) \quad \text {for }X=\mathrm {R},\,\mathrm {F} . \end{aligned}$$

Generating larger grids that include additional alternative central scale choices each with an additional dimension in the grid allows for the scale choice used in the convolution to be any arbitrary function of these independent central scales, $\mu _X = \mu _X(\mathcal {O}_1, \mathcal {O}_2, \ldots )$. The functionality for storing an additional central scale is implemented in fastNLO but entails an increase in the grid size and therefore also on the memory footprint during the computation. Using the short-hand notation

$$\begin{aligned} L^{[j]}_{\mathrm {X}}&\equiv \ln \left( \frac{\mu _X^2(\mu ^{[j]})}{\mu ^{2[j]}}\right) \quad \text {for }X=\mathrm {R},\,\mathrm {F}, \nonumber \\ \alpha _{\mathrm {s}} ^{[j_{\rightarrow \mathrm {R}}]}&\equiv \alpha _{\mathrm {s}} (\mu _{\mathrm{R}} (\mu ^{[j]})), { \text { and }} f^{[i,j_{\rightarrow \mathrm {F}}]}_{a} \equiv f_a(x^{[i]},\mu _{\mathrm{F}} (\mu ^{[j]})) , \end{aligned}$$

the full scale dependence up to NNLO is given by

16 $$\begin{aligned}&\sigma ^\text {NNLO}(\mu _{\mathrm{R}},\mu _{\mathrm{F}}) = \sum _{i,j} \biggl (\frac{\alpha _{\mathrm {s}} ^{[j_{\rightarrow \mathrm {R}}]}}{2\pi }\biggr )^{k} f^{[i,j_{\rightarrow \mathrm {F}}]}_{a} \; \hat{\sigma }^{(0)}_{a[i,j]} \nonumber \\&\quad +\sum _{i,j} \biggl (\frac{\alpha _{\mathrm {s}} ^{[j_{\rightarrow \mathrm {R}}]}}{2\pi }\biggr )^{k+1} \biggl \{ f^{[i,j_{\rightarrow \mathrm {F}}]}_{a} \; \hat{\sigma }^{(1)}_{a[i,j]}\nonumber \\&\qquad + \Bigl [ k \beta _0 f^{[i,j_{\rightarrow \mathrm {F}}]}_{a} L^{[j]}_{\mathrm {R}} \nonumber \\&\qquad \quad -(P^{(0)}\otimes f)^{[i,j_{\rightarrow \mathrm {F}}]}_{a} L^{[j]}_{\mathrm {F}} \Bigr ] \; \hat{\sigma }^{(0)}_{a[i,j]} \biggr \} \nonumber \\&\quad +\sum _{i,j} \biggl (\frac{\alpha _{\mathrm {s}} ^{[j_{\rightarrow \mathrm {R}}]}}{2\pi }\biggr )^{k+2} \biggl \{ f^{[i,j_{\rightarrow \mathrm {F}}]}_{a} \; \hat{\sigma }^{(2)}_{a[i,j]} \nonumber \\&\qquad + \Bigl [ (k+1) \beta _0 f^{[i,j_{\rightarrow \mathrm {F}}]}_{a} L^{[j]}_{\mathrm {R}} \nonumber \\&\qquad \quad -(P^{(0)}\otimes f)^{[i,j_{\rightarrow \mathrm {F}}]}_{a} L^{[j]}_{\mathrm {F}} \Bigr ] \; \hat{\sigma }^{(1)}_{a[i,j]} \nonumber \\&\quad + \Bigl [ \Bigl ( k \beta _1 + \tfrac{1}{2}k(k+1)\beta _0^2 L^{[j]}_{\mathrm {R}} \Bigr ) \; f^{[i,j_{\rightarrow \mathrm {F}}]}_{a} L^{[j]}_{\mathrm {R}} \nonumber \\&\qquad \quad -(P^{(1)}\otimes f)^{[i,j_{\rightarrow \mathrm {F}}]}_{a} L^{[j]}_{\mathrm {F}} \nonumber \\&\qquad \quad +\tfrac{1}{2}(P^{(0)}\otimes P^{(0)}\otimes f)^{[i,j_{\rightarrow \mathrm {F}}]}_{a} L^{2[j]}_{\mathrm {F}} \nonumber \\&\qquad \quad + \Bigl ( \tfrac{1}{2} \beta _0 L^{[j]}_{\mathrm {F}} -(k+1) \beta _0 L^{[j]}_{\mathrm {R}} \Bigr ) \nonumber \\&\qquad \qquad \quad \times (P^{(0)}\otimes f)^{[i,j_{\rightarrow \mathrm {F}}]}_{a} L^{[j]}_{\mathrm {F}} \Bigr ] \; \hat{\sigma }^{(0)}_{a[i,j]} \biggr \} \, . \end{aligned}$$

In APPLgrid, this summation is performed on the fly only if and when required, with the convolutions with the splitting functions $P^{(n)}$ performed using Hoppet [24].

As an alternative to the analytical reconstruction of the scales in Eq. (16), individual grids for the additional independent coefficients of the scale logarithms can be generated. This corresponds to the default strategy in the fastNLO library and the full scale dependence can be reconstructed through

17 $$\begin{aligned}&\sigma ^\text {NNLO}(\mu _{\mathrm{R}},\mu _{\mathrm{F}}) = \sum _{i,j} \biggl (\frac{\alpha _{\mathrm {s}} ^{[j_{\rightarrow \mathrm {R}}]}}{2\pi }\biggr )^{k} f^{[i,j_{\rightarrow \mathrm {F}}]}_{a} \; \hat{\sigma }^{(0|0,0)}_{a[i,j]} \nonumber \\&\quad +\sum _{i,j} \biggl (\frac{\alpha _{\mathrm {s}} ^{[j_{\rightarrow \mathrm {R}}]}}{2\pi }\biggr )^{k+1} f^{[i,j_{\rightarrow \mathrm {F}}]}_{a} \; \nonumber \\&\qquad \times \biggl \{ \hat{\sigma }^{(1|0,0)}_{a[i,j]} + L^{[j]}_{\mathrm {R}} \; \hat{\sigma }^{(1|1,0)}_{a[i,j]} + L^{[j]}_{\mathrm {F}} \; \hat{\sigma }^{(1|0,1)}_{a[i,j]} \biggr \} \nonumber \\&\quad +\sum _{i,j} \biggl (\frac{\alpha _{\mathrm {s}} ^{[j_{\rightarrow \mathrm {R}}]}}{2\pi }\biggr )^{k+2} f^{[i,j_{\rightarrow \mathrm {F}}]}_{a} \; \nonumber \\&\qquad \times \biggl \{ \hat{\sigma }^{(2|0,0)}_{a[i,j]} + L^{[j]}_{\mathrm {R}} \; \hat{\sigma }^{(2|1,0)}_{a[i,j]} + L^{[j]}_{\mathrm {F}} \; \hat{\sigma }^{(2|0,1)}_{a[i,j]} \nonumber \\&\qquad \quad + L^{2[j]}_{\mathrm {R}} \; \hat{\sigma }^{(2|2,0)}_{a[i,j]} + L^{2[j]}_{\mathrm {F}} \; \hat{\sigma }^{(2|0,2)}_{a[i,j]} \nonumber \\ {}&\qquad \quad + L^{[j]}_{\mathrm {R}} \; L^{[j]}_{\mathrm {F}} \; \hat{\sigma }^{(2|1,1)}_{a[i,j]} \biggr \} \, , \end{aligned}$$

where the grids are produced in analogy with Eq. (14) but using the decomposition of Eq. (4)

$$\begin{aligned} \hat{\sigma }^{(p|\alpha ,\beta )}_{a[i,j]}&\xrightarrow {\text {MC}} \sum _{m=1}^{M_p} E^y_i(x_m) \; E^\tau _j(\mu _{m}) \; w^{(p)}_{a;m} \; \mathrm {d}\hat{\sigma }^{(p|\alpha ,\beta )}_{a;m} \, . \end{aligned}$$

Using additional coefficient grids reduces the numerical complexity of the a posteriori convolutions involving the splitting functions and is faster for these terms but increases the number of summations over the grids for the full NNLO calculation from three to ten. The evaluation of these additional terms can be performed using the full expressions or they can be obtained numerically by evaluating the Monte Carlo weights for six independent scale pairs $(\mu _{\mathrm{R}},\mu _{\mathrm{F}})$ and solving a linear equation for the coefficients.

## The APPLfast project

The APPLfast project provides a library of code written in C++ with Fortran callable components. It is a lightweight interface used to bridge between the NNLOjet code and the specific code for booking and filling the grids themselves using either APPLgrid or fastNLO.

The basic structure for the filling of either grid technology is essentially the same, and as such, much of the functionality for the interface exists as common code that is used for filling both, with only the code that actually fills the weights needing to be specific to either technology. Efforts are under way to implement a common filling API for both fastNLO and APPLgrid, which will allow significantly more of the specific filling code to be shared.

A design principle, applied from the outset, was that the interface should be as unobtrusive as possible in the NNLOjet code, and should provide no additional performance overhead in terms of execution time when not filling a grid. When filling a grid, any additional overhead should be kept as low as possible. This is achieved by the use of a minimal set of hook functions that can be called from within the NNLOjet code itself and which can be left within the code with no impact on performance if the grid filling functionality is not required. The original proof-of-concept implementation accessed the required variables for the weights, scales and momentum fractions via the NNLOjet data structures directly, but following this it was decided to instead implement custom access functions that allow, e.g., for a full decomposition of the event weights as described by Eq. (4), thus enabling a more straightforward design for the filling code.

Each process in NNLOjet consists of a large number of subprocesses. In order to fill the grids, during the configuration stage the internal list of NNLOjet processes is mapped to a minimal set of the unique parton luminosities that are used for the grid. When filling, these internal NNLOjet process identifiers are used to determine which parton luminosity terms in the grid should be filled on the interface side.

Generating a cross section grid using NNLOjet typically involves four stages:

1. *Vegas adaption* This is the first stage in the standard NNLOjet workflow and is used to generate an optimised Vegas phase-space grid for the subsequent production runs. At this stage the grid filling is not enabled and NNLOjet can run in multi-threaded mode.
2. *Grid warm-up* This is required in order to optimise the limits for the phase space in *x* and $\mu _{\mathrm{F}}$ for the grids. During this stage, the NNLOjet code runs in a custom mode intended solely to sample the phase-space volume, thus skipping the costly evaluation of the Matrix Elements.
3. *Grid production* Here, the grids from stage 2 are filled with the weights generated from a full NNLOjet run, using the optimised phase-space sampling determined in stage 1. The calculation can be run in parallel using many independent jobs to achieve the desired statistical precision.
4. *Grid combination* In this stage, the grids from the individual jobs are combined, first merging the results for each of the LO, NLO (R and V), and NNLO (RR, VV, RV) terms separately, and subsequently assembling the respective grids into a final master grid.

The procedure to combine the interpolation grids closely follows the one developed for NNLOjet [25]. Each cross-section bin in the observable of each calculated grid is weighted with the same number as determined by the NNLOjet merging script for the combination of the final cross sections.

The stabilisation of higher-order cross sections with respect to statistical fluctuations demands a substantial number of events to be generated. This is particularly true for the double-real contribution, since the large number of final-state partons lead to a complex pattern of infrared divergences that need to be compensated. Typically, computing times of the order of hundreds of thousands of CPU hours are required. In stage 3 it is therefore mandatory to run hundreds to thousands of separate jobs in parallel, in particular for the NNLO sub-contributions. The resulting interpolation grids for each cross section and job typically are about 10–100 MBytes in size. The final master grid obtained by summing the output from all jobs then is somewhat larger than the largest single grid, because it contains at least one weight grid for each order in $\alpha _s$.

The interpolation accuracy must be evaluated to ensure that the results of the full calculation can be reproduced with the desired precision. For sufficiently well-behaved functions, as usually the case for PDFs, it is always possible to reach such precision by increasing the number of nodes in the fractional momentum *x* and scale $\mu$ at the cost of larger grid sizes. For proton-proton scattering, because of the additional momentum fraction associated with the second proton, the grid size grows quadratically with the number of *x* nodes.

**Fig. 1:**
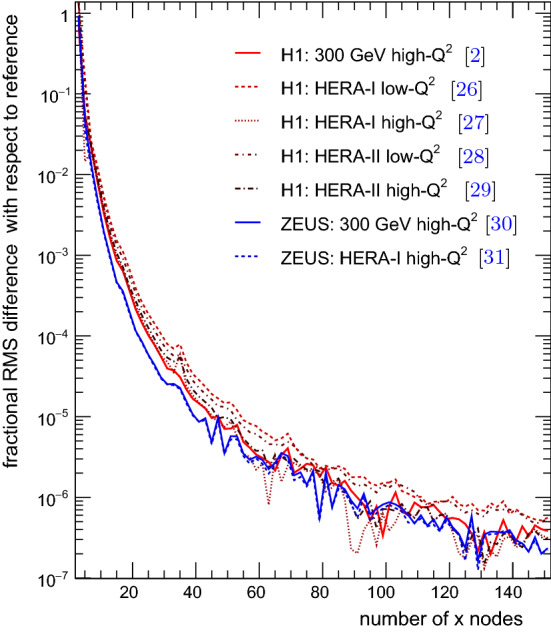
The RMS difference between the fast grid convolution and reference histogram as a function of the number of grid nodes in momentum fraction, *x* for the HERA inclusive jet measurements in DIS

To optimise the number of nodes necessary to achieve a sufficient approximation accuracy, several parameters and techniques can be adapted: Notably, the order or method of interpolation, the transform used for *x* and $\mu$, and the accessed ranges in *x* and $\mu$, as determined in the grid warm-up stage 3, can be chosen such that the number of nodes can be reduced significantly while retaining the same approximation accuracy. Figure 1 shows the root mean square (RMS) of the fractional difference of the fast grid convolution with respect to the corresponding reference for HERA inclusive jet production data. This uses a third order interpolation in the transformed *y*(*x*) variable and the transform from Eq. (10) and shows that the precision is better than one per mille for grids with 20 *x* nodes, and better than 0.1 per mille for grids with more than 30 *x* nodes.

**Fig. 2:**
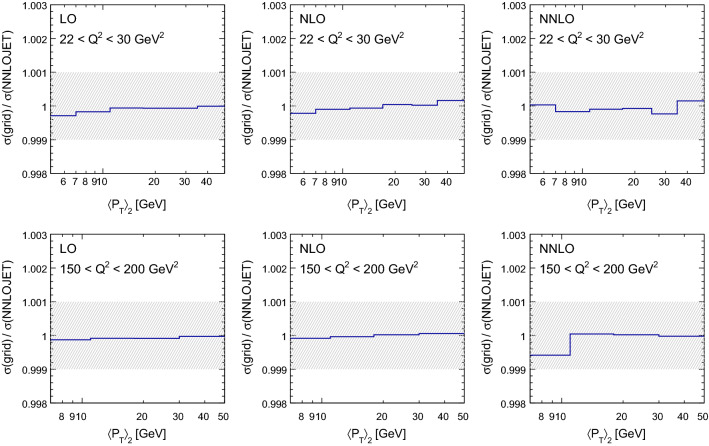
Validation of the grid accuracy in di-jet production at low-$Q^{2}$ ($22<Q^{2} <30\,\mathrm {GeV}^2$, top row) and high-$Q^{2}$ ($150< Q^{2} < 200\,\mathrm {GeV}^2$, bottom row). The shaded area indicates an agreement of 0.1%

For a specific process, observable, and phase space selection, an initial indication of the level of precision can be gained already using a single job by comparing the interpolated result with the reference calculation for the chosen PDF set for each bin in the observable.

Since identical events are filled both into the grid and into the reference cross section, then any statistical fluctuations should be reproduced and thus a limited number of events is usually sufficient for this validation. Subsequently, a similar level of precision should be possible for each of the contributions for the full calculation. In future, this could be exploited to avoid the time consuming access to the reference PDF during the full NNLOjet calculation itself during the mass production of interpolation grids at a previously validated level of precision.

For the grids presented here, all events have been produced with reference weights and the sufficiently accurate reproduction of the reference has been verified; for each of the individual output grids from the many separate runs for each contribution, for the combined grids from each contribution, and for the final overall grid combination. Figure 2 compares the fast convolution with the reference from NNLOjet for di-jet data at low $Q^2$ from H1 [28] and demonstrates an agreement better than the per mille level for all bins.

**Fig. 3:**
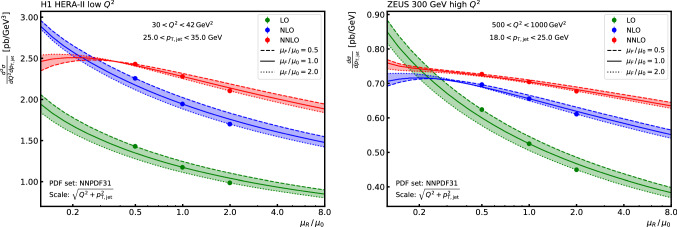
The scale dependence for a single bin in jet $p_\mathrm {T}$ with $25< p_\mathrm {T,jet} < 35\,\mathrm {GeV}$ for a range $30< Q^{2} < 42\,\mathrm {GeV}^2$ from H1 (left) and in jet $p_\mathrm {T}$ with $18< p_\mathrm {T,jet} < 25\,\mathrm {GeV}$ for a range $500< Q^{2} < 1000\,\mathrm {GeV}^2$ from ZEUS (right). The bands show the result of varying the factorisation scale $\mu _{\mathrm{F}}$ by factors between 0.5 and 2.0 with respect to the nominal scale. At each order three points indicate the result of symmetric variations of $\mu _{\mathrm{R}}$ and $\mu _{\mathrm{F}}$

Additional cross checks can be performed, for example, comparing the interpolated result of the final grid using an alternative PDF from the reference cross section, with an independent reference calculation for this same alternative PDF set. Here, of course, agreement can only be confirmed within the statistical precision of the two independent calculations. Moreover, it can be verified that the fast convolution with a change in scale, $\mu$, is consistent with the full calculation performed at that scale.

In addition, the independent and completely different scale variation techniques implemented in APPLgrid and fastNLO are cross-checked against each other and are found to agree. The resulting scale dependence with a choice for the nominal scale of $\mu _0^2 = Q^{2} +p_\mathrm {T,jet} ^2$, is illustrated in Fig. 3 for two bins in inclusive jet $p_\mathrm {T}$; one from the H1 low $Q^{2}$ data and one for the ZEUS high $Q^{2}$ data.

**Fig. 4:**
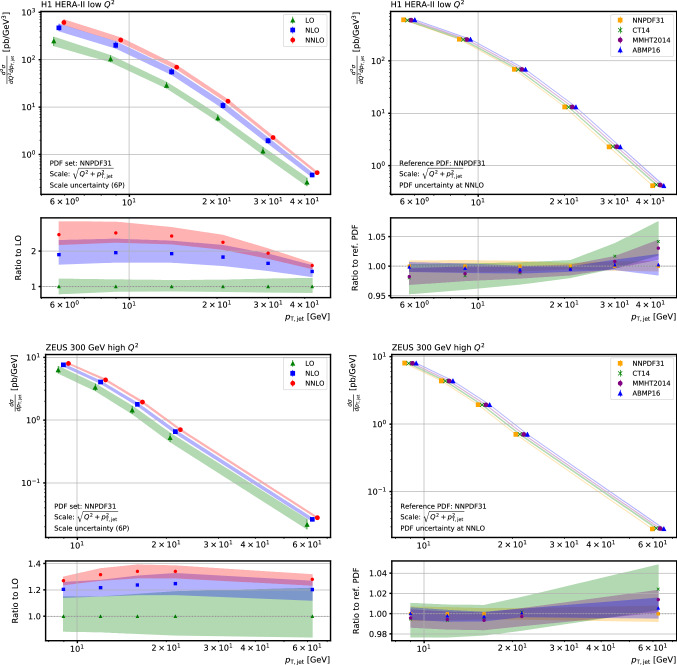
Inclusive jet cross section as a function of the jet $p_\mathrm {T}$ for two ranges in $Q^{2}$: $30< Q^{2} < 42\,\mathrm {GeV}^2$ for H1 data (upper row), and $500< Q^{2} < 1000\,\mathrm {GeV}^2$ for ZEUS data (lower row). On the left the LO, NLO, and NNLO predictions are shown using the NNPDF31 PDF set including their ratio to the LO in the respective lower panels. On the right the NNLO predictions are shown for the four PDF sets NNPDF31, CT14, MMHT2014, and ABMP16 including their ratio to the NNPDF31 PDF prediction in the respective lower panels. The bands indicate the uncertainty derived from six variations of the $\mu _{\mathrm{R}}$ and $\mu _{\mathrm{F}}$ scale factors as described in the text (left), respectively the PDF uncertainty as prescribed in the respective publications. For better visibility the points in all upper panels are slightly shifted in $p_\mathrm {T,jet}$

A significant benefit of using such interpolation grids is that the detailed uncertainties can be calculated without the need to rerun the calculation. This is illustrated in Fig. 4, which shows the full seven point scale variation and the PDF uncertainties derived for the $p_\mathrm {T,jet}$ dependent cross sections of the same H1 and ZEUS measurements from before. The seven point scale uncertainty is a conventional means of estimating the possible effect of uncalculated higher orders. It is defined by the maximal upward and downward changes in the cross section when varying the renormalisation and factorisation scales by factors of two around the nominal scale in the following six combinations of $(\mu _{\mathrm{R}}/\mu _0, \mu _{\mathrm{F}}/\mu _0)$: (1 / 2, 1 / 2), (2, 2), (1 / 2, 1), (1, 1 / 2), (2, 1), and (1, 2). The PDF uncertainties at the $1\,\sigma$ level are evaluated as prescribed for the respective PDF sets^1^: NNPDF31 [33], CT14 [34], MMHT2014 [35], and ABMP16 [36]. In all plots PDFs at NNLO have been used with $\alpha _{\mathrm {s}} (M_{{\mathrm {Z}}}) =0.118$.

## Application: determination of the strong coupling constant

As an application in using the DIS jet grids at NNLO, an extraction of the strong coupling constant, $\alpha _{\mathrm {s}} (M_{{\mathrm {Z}}})$, is performed using a fit of the NNLO QCD predictions from NNLOjet to the HERA inclusive jet cross-section data.

Seven sets of cross section measurements by the HERA experiments are considered for the $\alpha _{\mathrm {s}} (M_{{\mathrm {Z}}})$ determination: Five from H1 and two from ZEUS, each given by an inclusive jet cross section measurement as a function of $p_\mathrm {T,jet}$ and $Q^{2}$. The H1 results include measurements at $\sqrt{s}=300~\,\mathrm {GeV}$ [2] and $\sqrt{s}=320~\,\mathrm {GeV}$ [26–29], in the ranges $Q^{2} \lesssim 120~\,\mathrm {GeV}^2$ [26, 28] and $Q^{2} \gtrsim 120~\,\mathrm {GeV}^2$ [2, 27, 29], where jets are measured within a kinematic range between $4.5<p_\mathrm {T,jet} <80~\,\mathrm {GeV}$. For ZEUS, the data are similarly comprised of measurements at $\sqrt{s}=300~\,\mathrm {GeV}$ [30] and $\sqrt{s}=320~\,\mathrm {GeV}$ [31], but in the range $Q^{2} > 125~\,\mathrm {GeV}^2$ and with jets having $p_\mathrm {T,jet} >8~\,\mathrm {GeV}$. For all data sets jets are defined in the Breit frame of reference using the $k_T$ jet algorithm with a jet-resolution parameter $R=1$.

The methodology for the $\alpha _{\mathrm {s}} (M_{{\mathrm {Z}}})$ determination employs the same technique as Refs. [10] and [37]. In brief, a goodness-of-fit quantifier between data and prediction that depends on $\alpha _{\mathrm {s}} (M_{{\mathrm {Z}}})$ is defined in terms of a $\chi ^{2}$ function, which is based on normally-distributed relative uncertainties and accounts for all experimental, hadronisation, and PDF uncertainties. The experimental uncertainties, and the hadronisation corrections and their uncertainties are provided together with the data by the H1 and ZEUS collaborations. The PDF uncertainties are calculated using the prescriptions provided by the respective PDF fitting groups. The $\chi ^{2}$ function is then minimised using Minuit [38]. The $\alpha _{\mathrm {s}} (M_{{\mathrm {Z}}})$ dependence in the predictions takes into account the contributions from both the hard coefficients and the PDFs. The latter is evaluated using the DGLAP evolution as implemented in the Apfel++ package [39, 40], using the PDFs evaluated at a scale of $\mu _0=20~\,\mathrm {GeV}$. A different choice for the value of $\mu _0$ is found to have negligible impact on the results. The uncertainties on the fit quantity are obtained by the HESSE algorithm and validated by comparison with results obtained using the MINOS algorithm [38]. The uncertainties are separated into experimental (exp), hadronisation (had), and PDF uncertainties (PDF) by repeating the fit excluding uncertainty components.

Following Ref. [10], a representative value is assigned for the renormalisation scale to each single data cross section measurement denoted by $\tilde{\mu }$. This is determined from the lower and upper bin boundaries in $Q^{2}$ and $p_\mathrm {T,jet}$ (denoted with subscripts *dn* and *up*) as

18 $$\begin{aligned} \tilde{\mu }^2 = \sqrt{Q^{2}_{\mathrm{dn}} Q^{2}_{\mathrm{up}}} + p^{\mathrm{jet}}_{\mathrm{T,dn}}p^{\mathrm{jet}}_{\mathrm{T,up}}. \end{aligned}$$

The calculation is performed using five massless flavours, and as such, for the $\alpha _{\mathrm {s}}$ fit, the data are restricted to be above twice the mass of the *b*-quark [41], i.e. $\tilde{\mu }>2m_b$.

The nominal predictions are obtained using the NNPDF3.1 PDF set [33], which is used to further define the PDF and PDF$\alpha _{\mathrm {s}}$ uncertainties. The PDFset uncertainties, on the other hand, are determined by separately repeating the $\alpha _{\mathrm {s}}$ fit using predictions at NNLO that are evaluated using the ABMP [36], CT14 [34], HERAPDF2.0 [42], MMHT [35], and NNPDF3.1 PDF sets. The exact definition of the PDF$\alpha _{\mathrm {s}}$ and PDFset uncertainties can be found in Ref. [37].

**Table 1:** A summary of values of $\alpha _{\mathrm {s}} (M_{{\mathrm {Z}}})$ from fits to HERA inclusive jet cross section measurements using NNLO predictions. The uncertainties denote the experimental (exp), hadronisation (had), PDF, PDF$\alpha _{\mathrm {s}}$, PDFset and scale uncertainties as described in the text. The rightmost three columns denote the quadratic sum of the theoretical uncertainties (th), the total (tot) uncertainties and the value of $\chi ^{2}/n_\mathrm {dof}$ of the corresponding fit

Data	$\tilde{\mu } _{\mathrm{cut}}$	$\alpha _{\mathrm {s}} (M_{{\mathrm {Z}}})$ with uncertainties	th	tot	$\chi ^{2}/n_\mathrm {dof}$
**H1 inclusive jets**${}^\dagger$					
$300\,\,\mathrm {GeV}$ high-$Q^{2}$	$2m_b$	$0.1217\,(31)_{\mathrm{exp}}\,(22)_{\mathrm{had}}\,(5)_{\mathrm{PDF}}\,(3)_{\mathrm{PDF\alpha _{\mathrm {s}}}}\,(5)_{\mathrm{PDFset}}\,(35)_{\mathrm{scale}}$	$(42)_{\mathrm{th}}$	$(52)_{\mathrm{tot}}$	5.6 / 15
HERA-I low-$Q^{2}$	$2m_b$	$0.1093\,(17)_{\mathrm{exp}}\,(8)_{\mathrm{had}}\,(5)_{\mathrm{PDF}}\,(5)_{\mathrm{PDF\alpha _{\mathrm {s}}}}\,(7)_{\mathrm{PDFset}}\,(33)_{\mathrm{scale}}$	$(35)_{\mathrm{th}}$	$(39)_{\mathrm{tot}}$	17.5 / 22
HERA-I high-$Q^{2}$	$2m_b$	$0.1136\,(24)_{\mathrm{exp}}\,(9)_{\mathrm{had}}\,(6)_{\mathrm{PDF}}\,(4)_{\mathrm{PDF\alpha _{\mathrm {s}}}}\,(4)_{\mathrm{PDFset}}\,(28)_{\mathrm{scale}}$	$(31)_{\mathrm{th}}$	$(39)_{\mathrm{tot}}$	15.5 / 23
HERA-II low-$Q^{2}$	$2m_b$	$0.1187\,(18)_{\mathrm{exp}}\,(8)_{\mathrm{had}}\,(4)_{\mathrm{PDF}}\,(4)_{\mathrm{PDF\alpha _{\mathrm {s}}}}\,(3)_{\mathrm{PDFset}}\,(45)_{\mathrm{scale}}$	$(46)_{\mathrm{th}}$	$(50)_{\mathrm{tot}}$	29.6 / 40
HERA-II high-$Q^{2}$	$2m_b$	$0.1126\,(19)_{\mathrm{exp}}\,(9)_{\mathrm{had}}\,(6)_{\mathrm{PDF}}\,(4)_{\mathrm{PDF\alpha _{\mathrm {s}}}}\,(2)_{\mathrm{PDFset}}\,(32)_{\mathrm{scale}}$	$(34)_{\mathrm{th}}$	$(39)_{\mathrm{tot}}$	34.7 / 29
**ZEUS inclusive jets**					
$300\,\,\mathrm {GeV}$ high-$Q^{2}$	$2m_b$	$0.1213\,(28)_{\mathrm{exp}}\,(3)_{\mathrm{had}}\,(5)_{\mathrm{PDF}}\,(2)_{\mathrm{PDF\alpha _{\mathrm {s}}}}\,(3)_{\mathrm{PDFset}}\,(26)_{\mathrm{scale}}$	$(27)_{\mathrm{th}}$	$(39)_{\mathrm{tot}}$	28.6 / 29
HERA-I high-$Q^{2}$	$2m_b$	$0.1181\,(27)_{\mathrm{exp}}\,(16)_{\mathrm{had}}\,(6)_{\mathrm{PDF}}\,(2)_{\mathrm{PDF\alpha _{\mathrm {s}}}}\,(6)_{\mathrm{PDFset}}\,(25)_{\mathrm{scale}}$	$(31)_{\mathrm{th}}$	$(41)_{\mathrm{tot}}$	20.8 / 29
**H1 inclusive jets**${}^\dagger$					
H1 inclusive jets	$2m_b$	$0.1133\,(10)_{\mathrm{exp}}\,(6)_{\mathrm{had}}\,(5)_{\mathrm{PDF}}\,(4)_{\mathrm{PDF\alpha _{\mathrm {s}}}}\,(2)_{\mathrm{PDFset}}\,(39)_{\mathrm{scale}}$	$(40)_{\mathrm{th}}$	$(41)_{\mathrm{tot}}$	125.8 / 133
H1 inclusive jets	$28\,\,\mathrm {GeV}$	$0.1153\,(19)_{\mathrm{exp}}\,(9)_{\mathrm{had}}\,(2)_{\mathrm{PDF}}\,(2)_{\mathrm{PDF\alpha _{\mathrm {s}}}}\,(3)_{\mathrm{PDFset}}\,(26)_{\mathrm{scale}}$	$(28)_{\mathrm{th}}$	$(33)_{\mathrm{tot}}$	44.1 / 60
**ZEUS inclusive jets**					
ZEUS inclusive jets	$2m_b$	$0.1199\,(20)_{\mathrm{exp}}\,(8)_{\mathrm{had}}\,(6)_{\mathrm{PDF}}\,(1)_{\mathrm{PDF\alpha _{\mathrm {s}}}}\,(5)_{\mathrm{PDFset}}\,(26)_{\mathrm{scale}}$	$(29)_{\mathrm{th}}$	$(35)_{\mathrm{tot}}$	49.8 / 59
ZEUS inclusive jets	$28\,\,\mathrm {GeV}$	$0.1194\,(24)_{\mathrm{exp}}\,(7)_{\mathrm{had}}\,(6)_{\mathrm{PDF}}\,(1)_{\mathrm{PDF\alpha _{\mathrm {s}}}}\,(5)_{\mathrm{PDFset}}\,(25)_{\mathrm{scale}}$	$(27)_{\mathrm{th}}$	$(34)_{\mathrm{tot}}$	39.3 / 43
**HERA inclusive jets**					
HERA inclusive jets	$2m_b$	$0.1149\,(9)_{\mathrm{exp}}\,(5)_{\mathrm{had}}\,(4)_{\mathrm{PDF}}\,(3)_{\mathrm{PDF\alpha _{\mathrm {s}}}}\,(2)_{\mathrm{PDFset}}\,(37)_{\mathrm{scale}}$	$(38)_{\mathrm{th}}$	$(39)_{\mathrm{tot}}$	182.9 / 193
HERA inclusive jets	$28\,\,\mathrm {GeV}$	$0.1170\,(15)_{\mathrm{exp}}\,(7)_{\mathrm{had}}\,(3)_{\mathrm{PDF}}\,(2)_{\mathrm{PDF\alpha _{\mathrm {s}}}}\,(3)_{\mathrm{PDFset}}\,(24)_{\mathrm{scale}}$	$(25)_{\mathrm{th}}$	$(29)_{\mathrm{tot}}$	85.7 / 104

${}^\dagger$Previously fit in Ref. [10]

**Fig. 5:**
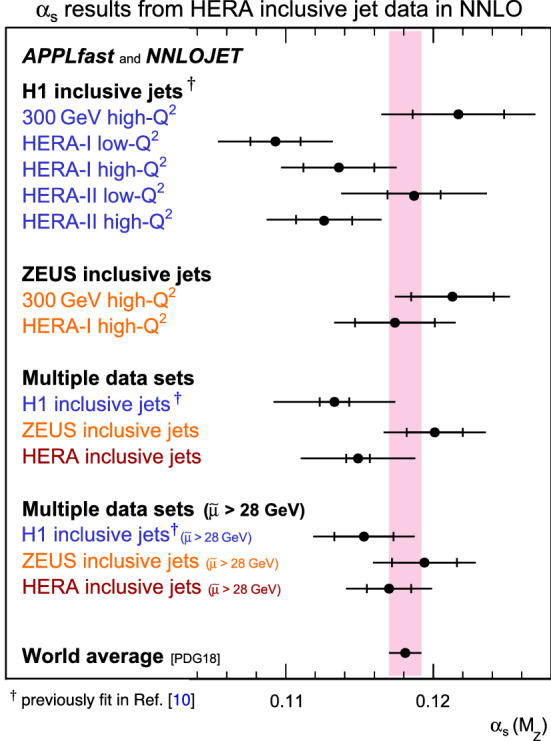
Summary of $\alpha _{\mathrm {s}} (M_{{\mathrm {Z}}})$ values in comparison with the world average value. The inner error bars indicate experimental uncertainties, and the full errors the total uncertainty, comprised of the experimental and theoretical uncertainties. The lower set of values represent fits to data restricted to $\tilde{\mu }>28\,\,\mathrm {GeV}$

Results for the values of $\alpha _{\mathrm {s}} (M_{{\mathrm {Z}}})$ as obtained from the individual fits to the inclusive jet cross section data are collected in Table 1. The entries for the H1 data sets correspond to values previously reported in Ref. [10] but some have been updated using NNLO predictions with higher statistical precision. New results are presented for the fits to the ZEUS inclusive jet cross section data [30, 31] and fits to all the H1 and ZEUS inclusive jet cross section data, which are the principle results of this current study. The $\alpha _{\mathrm {s}} (M_{{\mathrm {Z}}})$ values from the individual data sets are found to be mutually compatible within their respective errors. Figure 5 summarises the values for a visual comparison, and includes the world average [41, 43], which is seen to be consistent with the value extracted here. All the H1 and ZEUS inclusive jet cross section data are found to be in good agreement with the NNLO predictions, as indicated by the individual $\chi ^{2}/n_\mathrm {dof}$ values in Table 1. From the fit to all HERA inclusive jet data a value of $\alpha _{\mathrm {s}} (M_{{\mathrm {Z}}}) =0.1149\,(9)_{\mathrm{exp}}\,(38)_{\mathrm{th}}$ is obtained, where *exp* and *th* denote the experimental and theoretical uncertainties, respectively, and where the latter is obtained by combining individual theory uncertainties in quadrature. A detailed description of the uncertainty evaluation procedure can be found in Ref. [10]. The fit yields $\chi ^{2}/n_\mathrm {dof} =182.9/193$, thus indicating an excellent description of the data by the NNLO predictions. Furthermore, an overall high degree of consistency for all of the HERA inclusive jet cross section data is found.

The dominant uncertainty in the extraction of $\alpha _{\mathrm {s}}$ arises from the renormalisation scale dependence of the NNLO predictions. As such, the fits are repeated with a restricted data selection requiring $\tilde{\mu }>28~\,\mathrm {GeV}$, chosen in order to obtain a balance between the experimental uncertainty from the measurements and the scale dependence from the theory predictions and so reduce the total uncertainty on the final extraction. It was verified that the extracted $\alpha _{\mathrm {s}}$ value and the associated uncertainty are stable with respect to variations of $\tilde{\mu }$ around $28~\,\mathrm {GeV}$. This fit represents the primary result and the value of $\alpha _{\mathrm {s}} (M_{{\mathrm {Z}}})$ is determined to be

19 $$\begin{aligned} \alpha _{\mathrm {s}} (M_{{\mathrm {Z}}}) = 0.1170\,(15)_\text {exp}\,(25)_\text {th}, \end{aligned}$$

with the uncertainty decomposition given in Table 1. The value is found to be consistent with the world average within uncertainties. The obtained uncertainties are competitive with other determinations from a single observable.

**Fig. 6:**
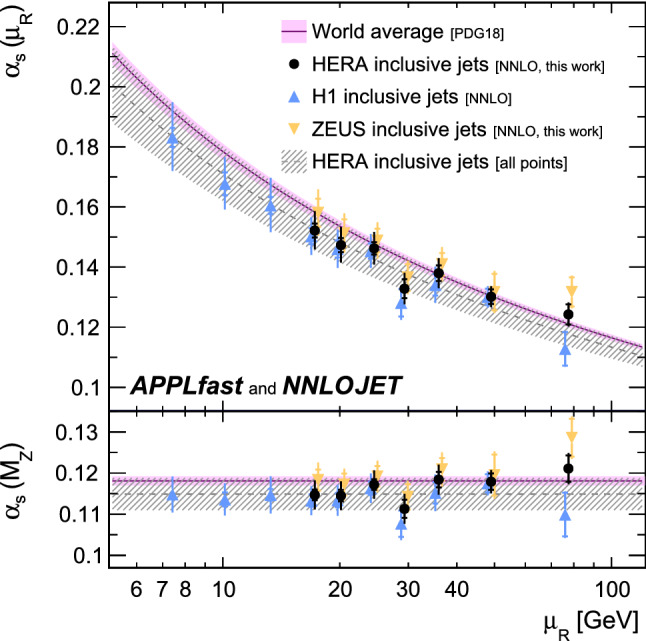
Results for $\alpha _{\mathrm {s}} (M_{{\mathrm {Z}}})$ (lower panel) and corresponding values for $\alpha _{\mathrm {s}} (\mu _{\mathrm{R}})$ (upper panel) from fits to inclusive jet data points arranged in groups of similar $\mu _{\mathrm{R}}$. The upper panel is obtained by applying the expectation from the QCD renormalisation group equation, as it also enters the NNLO predictions. The inner error bars indicate experimental uncertainties, and the full error bars the total uncertainty. The upper triangles show results from H1 data, which were previously fit in Ref. [10] and are here partially updated with NNLO predictions with higher statistical accuracy. The lower triangles indicate the new results from ZEUS data. The full circles show the combined results from H1 and ZEUS data taken together and are labeled HERA inclusive jets. The shaded band indicates the world average value with its uncertainty, and the dashed line and hatched band indicate the result obtained from the fit to all inclusive jet data and its uncertainty

**Table 2:** Values of the strong coupling constant at the ${\mathrm {Z}}$-boson mass, $\alpha _{\mathrm {s}} (M_{{\mathrm {Z}}})$, obtained from fits to groups of data with comparable values of $\mu _{\mathrm{R}}$. The first (second) uncertainty of each point corresponds to the experimental (theory) uncertainties. The theory uncertainties include PDF related uncertainties and the dominating scale uncertainty

$\mu _{\mathrm{R}}$	H1	ZEUS	HERA
(GeV)	$\alpha _{\mathrm {s}} (M_{{\mathrm {Z}}})$	$\alpha _{\mathrm {s}} (M_{{\mathrm {Z}}})$	$\alpha _{\mathrm {s}} (M_{{\mathrm {Z}}})$
7.4	$0.1148\,(12)\,(42)$	$-$	$0.1148\,(12)\,(42)$
10.1	$0.1136\,(17)\,(35)$	$-$	$0.1136\,(17)\,(35)$
13.3	$$ 0.1147\,(14)\,(43)$$	$$ -$$	$$ 0.1147\,(14)\,(43)$$
17.2	$0.1133\,(15)\,(32)$	$0.1183\,(26)\,(34)$	$0.1147\,(13)\,(33)$
20.1	$0.1134\,(17)\,(34)$	$0.1172\,(27)\,(28)$	$0.1145\,(14)\,(32)$
24.5	$0.1163\,(16)\,(32)$	$0.1192\,(25)\,(29)$	$0.1172\,(13)\,(32)$
29.3	$0.1077\,(32)\,(34)$	$0.1142\,(31)\,(24)$	$0.1113\,(22)\,(29)$
36.0	$0.1152\,(26)\,(36)$	$0.1209\,(28)\,(31)$	$0.1184\,(19)\,(31)$
49.0	$0.1175\,(22)\,(19)$	$0.1195\,(50)\,(29)$	$0.1179\,(20)\,(20)$
77.5	$0.1099\,(53)\,(20)$	$0.1286\,(46)\,(24)$	$0.1211\,(32)\,(20)$

The running of $\alpha _{\mathrm {s}} (\mu _{\mathrm{R}})$ can be inferred from separate fits to groups of data points that share a similar value of the renormalisation scale, as estimated by $\tilde{\mu }$ in Eq. (18). To this end, the $\alpha _{\mathrm {s}} (M_{{\mathrm {Z}}})$ values are determined for each $\tilde{\mu }$ collection individually, and are summarised in Table 2 and shown in the bottom panel of Fig. 6. All values are mutually compatible and in good agreement with the world average, and no significant dependence on $\mu _{\mathrm{R}}$ is observed. The corresponding values for $\alpha _{\mathrm {s}} (\mu _{\mathrm{R}})$, as determined using the QCD renormalisation group equation, are displayed in the top panel of Fig. 6, illustrating the running of the strong coupling. The dashed line corresponds to the prediction for the $\mu _{\mathrm{R}}$ dependence using the $\alpha _{\mathrm {s}}$ value of Eq. (19). The predicted running is in excellent agreement with the individual $\alpha _{\mathrm {s}} (\mu _{\mathrm{R}})$ determinations, further reflecting the internal consistency of the study.

To conclude this study it is worth commenting on the robustness of the procedure. On the theory side, the inclusive jet cross section represents an observable that is well defined in perturbative QCD and only moderately affected by non-perturbative effects and experimentally, this study rests on a solid basis, making use of measurements from two different experiments based on three separate data taking periods, which cover two different centre-of-mass energies and two kinematic regions in $Q^{2}$. As a result, although only a single observable is used in the determination of $\alpha _{\mathrm {s}}$, a highly competitive experimental and theoretical precision is achieved.

## Conclusions and outlook

NNLO calculations in perturbative QCD are rapidly becoming the new standard for many important scattering processes. These calculations are critical in reducing theory uncertainties and often improve the description of the increasingly precise data, sometimes even resolving prior tensions. However, the computational resources required for such calculations prohibit their use in applications that require a frequent re-evaluation using different input conditions, e.g. fitting procedures for PDFs and Standard Model parameters.

Fast interpolations grid techniques circumvent these limitations by allowing for the a posteriori interchange of PDFs, values of the strong coupling $\alpha _{\mathrm {s}}$, and scales in the prediction at essentially no cost. In this article the APPLfast project is discussed, which provides a generic interface for the APPLgrid and fastNLO grid libraries to produce interpolation tables where the hard coefficient functions are computed by the NNLOjet program. Details on the extension of the techniques to NNLO accuracy and their implementation for DIS are discussed, together with the public release of NNLO grid tables for jet cross-section measurements at HERA [9].

As an application of the grids, an extraction of the strong coupling constant $\alpha _{\mathrm {s}}$ has been performed, based on jet data at HERA, closely following the methodology in Refs. [10, 37]. In contrast to Ref. [10], where the $\alpha _{\mathrm {s}}$ determination considered both inclusive and di-jet cross section data from H1 alone, this current analysis includes data from both the H1 and ZEUS experiments, but $\alpha _{\mathrm {s}}$ is fitted solely using the single jet inclusive data. The usage of a single observable facilitates the simultaneous determination of $\alpha _{\mathrm {s}} (M_{{\mathrm {Z}}})$ from two experiments, as the observable is defined identically between both experiments and thus reduces ambiguities in the treatment of theory uncertainties. This work represents one of the first determinations of the strong coupling constant to include both H1 and ZEUS DIS jet data at NNLO accuracy, where such a determination is only possible using the foundational work presented in this paper. The determination of $\alpha _{\mathrm {s}} (M_{{\mathrm {Z}}})$ from H1 and ZEUS data taken together provides a best-fit value of $\alpha _{\mathrm {s}} (M_{{\mathrm {Z}}}) = 0.1170\,(15)_\text {exp}\,(25)_\text {th}$.

Although the discussion in the present work was limited to the DIS process, the implementation in both APPLfast and NNLOjet is fully generic and thus generalisable to hadron-hadron collider processes. This means that all NNLO calculations available from within NNLOjet , such as di-jet production and $V+\text {jet}$ production in proton-proton scattering, are interfaced to grid-filling tools in a rather straightforward manner. This generalisation will be presented in a future publication.

## Data Availability

This manuscript has no associated data or the data will not be deposited. [Authors’ comment: The data generated in the context of this publication is comprised of the fast interpolation grids at NNLO accuracy. They are publicly available on the designated platform at ploughshare.web.cern.ch and can be freely downloaded and used to reproduce all results from the manuscript.]

## Footnotes

1 The full LHAPDF [32] names for each of the sets are: NNPDF31_nnlo_as_0118, CT14nnlo, MMHT2014nnlo68cl, and ABMP16als118_5_nnlo respectively.
